# Gestational age references and trajectory of fetal haemoglobin in relation to outcome in neonates born before 30 weeks of gestation: a single-centre retrospective cohort study in The Netherlands

**DOI:** 10.1016/j.eclinm.2026.104256

**Published:** 2026-10-02

**Authors:** Jip H. van Daelen, Norani H. Gangaram-Panday, Elise J. Huisman, H. Rob Taal, Thomas R.L. Klei, Enrico Lopriore, Irwin K.M. Reiss, Andrei N. Tintu, Pauline M. Snijder

**Affiliations:** a Division of Neonatology, Department of Neonatal & Paediatric Intensive Care, Erasmus MC Sophia Children's Hospital, Erasmus Medical University Centre, Rotterdam, the Netherlands; b Division of Paediatric Haematology, Department of Paediatrics, Erasmus MC Sophia Children's Hospital, Erasmus Medical University Centre, Rotterdam, the Netherlands; c Department of Medical Affairs, Unit of Transfusion Medicine, Sanquin Blood Bank, Amsterdam, the Netherlands; d Department of Product and Process Development, Sanquin Blood Supply, Amsterdam, the Netherlands; e Division of Neonatology, Department of Paediatrics, Leiden University Medical Centre, Leiden, the Netherlands; f University Children's Hospital, Division of Neonatology and Paediatric Intensive Care, University Medical Centre Hamburg-Eppendorf, Hamburg, Germany; g Department of Clinical Chemistry Rotterdam, Erasmus Medical Centre, Zuid-Holland, the Netherlands

**Keywords:** Fetal haemoglobin, Extremely preterm neonates, Red blood cell transfusion, Gestational age, Prematurity related disease

## Abstract

**Background:**

Fetal haemoglobin (HbF) has a high oxygen affinity and predominates during fetal life. After birth, HbF is progressively replaced by adult haemoglobin (HbA). In preterm neonates, transfusion with HbA-containing red blood cells (RBCs) may accelerate this transition, increasing tissue oxygen exposure and oxidative stress, which have been associated with retinopathy of prematurity, bronchopulmonary dysplasia, and necrotizing enterocolitis. We aimed to establish gestational age (GA)-specific reference values and examine postnatal HbF trajectories in relation to transfusion exposure and outcomes, focusing on neonates born before 30 weeks’ gestation.

**Methods:**

In this single-centre retrospective cohort study, 856 neonates with HbF measurements (24–42 weeks' gestation) admitted to the ErasmusMC NICU, Rotterdam, the Netherlands (1 February 2020–1 February 2024) were included. GA-specific HbF reference values were derived from day 0 samples. Longitudinal HbF trajectories during NICU admission were analysed in 458 neonates born before 30 weeks’ gestation using 9461 blood gas samples. Linear mixed-effects models assessed associations between HbF decline, transfusion exposure, and prematurity-related outcomes.

**Findings:**

HbF at birth remained high until approximately 35 weeks' gestation (median ≥0.85) and declined towards term. In neonates born before 30 weeks' gestation, HbF declined during the first postnatal weeks, with the steepest decline seen in babies born at 24–25 weeks’ gestation. Neonates who received RBC transfusions showed a steeper HbF decline than those who did not (p < 0.001), as did neonates with prematurity-related outcomes compared with those without major morbidity or mortality (p < 0.001).

**Interpretation:**

RBC transfusions accelerate HbF decline in neonates born before 30 weeks’ gestation. Its association with prematurity-related morbidity supports evaluation of HbF-preserving transfusion strategies. Future studies should incorporate the interval and frequency of successive transfusions through time-varying cumulative transfusion exposure or analyses centred on individual transfusion events to further refine the temporal dynamics of HbF decline and recovery. These studies should ultimately evaluate the optimal interval and frequency of transfusions and whether HbF-preserving transfusion strategies improve clinical outcomes in extremely preterm neonates.

**Funding:**

No funding was received for this work.

**Research in context**

**Evidence before this study**

We searched PubMed for studies assessing fetal haemoglobin (HbF) trajectories, red blood cell (RBC) transfusions, and prematurity-related outcomes in preterm neonates published from database inception to August 18, 2026, without language restrictions. The search strategy combined the terms “fetal hemoglobin”, “fetal haemoglobin”, or “HbF” with terms related to prematurity (“preterm”, “premature”, “neonate”) and HbF trajectories or clinical outcomes (“trajectory”, “longitudinal”, “transfusion”, “retinopathy of prematurity”, “bronchopulmonary dysplasia”, “necrotizing enterocolitis”, “necrotising enterocolitis”). Existing evidence shows that HbF declines rapidly after birth in preterm neonates and that transfusion with adult donor RBCs may accelerate this transition. Several observational studies have suggested associations between low HbF levels or rapid HbF decline and prematurity-related diseases such as retinopathy of prematurity and bronchopulmonary dysplasia. However, knowledge in this field has largely been based on relatively small cohorts, single time-point measurements, or studies without comprehensive longitudinal modelling. Gestational age–specific HbF reference values derived from contemporary large cohorts are scarce, and few studies have combined reference data, trajectory modelling, and clinical outcome analysis within a single study. More recently, the randomized BORN trial demonstrated that cord blood-derived RBC transfusions preserve HbF levels in extremely preterm neonates and the per-protocol analyses reported encouraging clinical findings supporting further evaluation of HbF-preserving transfusion strategies.

**Added value of this study**

In this large contemporary cohort of neonates born between 24 and 42 weeks' gestation, we establish gestational age–specific HbF reference values at birth using day 0 measurements from routine clinical care. In a cohort of preterm neonates born before 30 weeks' gestation with repeated HbF measurements, we model postnatal HbF trajectories using mixed-effects analysis. We demonstrate that HbF decline differs by degree of prematurity and that accelerated decline is associated with transfusion exposure and severe prematurity-related disease. By integrating reference data, longitudinal modelling, and outcome assessment, our study provides a comprehensive framework for interpreting HbF dynamics in neonates born before 30 weeks’ gestation.

**Implications of all the available evidence**

HbF trajectories in preterm neonates born before 30 weeks’ gestation are clinically relevant and differ substantially by gestational age and transfusion exposure. Establishing gestational age–specific reference values improves interpretation of HbF levels in research and clinical practice. The effect of the interval between successive transfusions on HbF dynamics warrants further investigation. The optimal timing and frequency of transfusions remain to be established. This could be addressed by incorporating time-varying cumulative transfusion exposure or analyses centred on individual transfusion events to further refine the temporal dynamics of HbF decline and recovery. Future studies should determine whether interventions aimed at preserving physiological HbF levels can reduce prematurity-related morbidity and improve outcomes.

## Introduction

Preterm neonates are exposed to the extrauterine environment before their organs have fully matured. Neonatal intensive care is aimed at recreating intrauterine conditions as closely as possible to support organ development. The development of organs depends on a balanced supply of oxygen, as both hypoxia and hyperoxia, may have detrimental effects on neonatal clinical outcomes.^1^, ^2^, ^3^, ^4^, ^5^

Oxygen delivery to tissues is mediated by the oxygen-carrying protein haemoglobin (Hb) in red blood cells (RBCs). During fetal life and the neonatal period, Hb of foetuses and neonates consist predominantly of fetal haemoglobin (HbF), with two α- and two γ-chains. HbF has a high oxygen affinity and a low affinity for 2,3-bisphosphoglycerate (2,3-BPG), shifting the oxygen-Hb dissociation curve to the left.^6^ This enables the transfer of oxygen from the maternal to the fetal circulation; the low oxygen saturated environment. After term birth, HbF is gradually replaced by adult haemoglobin (HbA), consisting of two α- and two β-chains. HbA has a higher affinity for 2,3-BPG, thereby facilitating oxygen release to tissues.^6^ By 12 months of age, HbA typically accounts for 98% of total Hb in children.^7^ Although HbF physiology and developmental Hb switching are well described, considerably less is known about the postnatal HbF trajectory following preterm birth, when neonates are exposed to a markedly different extrauterine environment. Contemporary gestational age (GA)-specific reference values at birth and longitudinal postnatal HbF trajectories following preterm birth therefore remain limited.

Very preterm neonates often receive RBC transfusions which are derived from adult donors and therefore contain mainly HbA. Frequent RBC transfusions in preterm neonates will lead to a transfusion-induced replacement of HbF by HbA, forcing the dissociation curve to shift to the right in a short time-interval, resulting in an increased oxygen delivery to immature tissues in comparison with HbF.^8^ As these tissues are not yet well-equipped to manage excessive free oxygen radicals, increased oxygen release may cause oxidative stress.^9^ Both a sharp decline in HbF and RBC transfusions have been associated, in observational studies, with the development of the prematurity related diseases such as retinopathy of prematurity (ROP), bronchopulmonary dysplasia (BPD) and, to a lesser extent, necrotizing enterocolitis (NEC).^2^, ^3^, ^4^^,^^10^, ^11^, ^12^, ^13^

These results have been the start of a growing interest in HbF-preserving strategies, as prevention of transfusion-induced HbF decline may positively impact the outcome of these already vulnerable preterm neonates. One such novel strategy proposed, is the development of umbilical cord blood-derived RBCs from healthy term neonates, which contain high levels of HbF. However, implementation of such strategies requires a clear understanding of physiological HbF levels and postnatal dynamics, which remain incompletely characterised, particularly in a gestational age–specific and longitudinal context following preterm birth. Therefore, the aim of this study is to establish GA –specific reference values for HbF in neonates across 24–42 weeks' gestation, and to subsequently analyse postnatal HbF trajectories in neonates born before 30 weeks’ gestation in relation to RBC transfusions and prematurity-related diseases.

## Methods

### Study design and population

We conducted a single-centre, retrospective observational study in neonates born between 24 and 42 weeks of gestation who were admitted to the NICU of the Sophia Children's Hospital, Erasmus University Medical Centre (Rotterdam, the Netherlands), between 1 February 2020 and 1 February 2024, and had available HbF measurements. HbF measurements were obtained as part of routine blood gas analyses performed during clinical care rather than for specific clinical indications, thereby limiting the risk of selection bias related to HbF measurement availability. This study is reported in accordance with the Strengthening the Reporting of Observational Studies in Epidemiology (STROBE) guidelines.

To establish GA-specific reference values for HbF we analysed HbF measurements obtained within 24 h after birth (postnatal day 0) in neonates born between 24 and 42 weeks’ gestation who had not received an RBC transfusion before HbF measurement.

To study postnatal HbF trajectory and its association with prematurity related diseases, we evaluated the postnatal HbF course in the group of neonates born before 30 weeks GA with at least one HbF measurement. Data were obtained from the electronic patient file and included the following patient characteristics: GA (in weeks), birth weight (grams), sex, Apgar score at 5 min, mode of delivery, length of NICU stay, number of RBC transfusions between NICU admission and last available HbF measurement, the prematurity related diseases ROP, BPD, NEC, and death before discharge. RBC transfusions were counted until the last available HbF measurement; consequently, the total number of transfusions during the complete NICU stay may be higher. Race and ethnicity were not routinely collected in the neonatal database and were therefore unavailable for inclusion in the analyses.

The following definitions of the investigated prematurity related diseases were used: ROP was diagnosed and classified based on the Dutch national guideline^14^ in line with the International Classification of ROP (ICROP2), which categorizes ROP severity in five stages plus an additional plus disease if present, as well as location of retinal vascular outgrowth in zones I–III (ICROP2, 2005).^15^ Severe ROP was defined as any case of ROP treated with laser or anti-VEGF therapy. Severe BPD was defined as the need for oxygen supplementation or ventilatory support at 36 weeks postmenstrual age (PMA), in line with established criteria, including an oxygen requirement of ≥30% and/or the need for positive pressure support.^16^^,^^17^ Severe NEC was defined as stage ≥ II according to the modified Bell's criteria.^18^ For each outcome analysis, neonates were classified independently according to the presence or absence of the respective outcome. Consequently, infants who developed multiple prematurity-related complications contributed to each relevant outcome analysis. The composite outcome was defined as the occurrence of one or more of severe ROP, severe BPD, severe NEC, or death before discharge.

### Ethics

The study received approval from the Medical Ethical Committee of the Erasmus Medical Centre, The Netherlands under reference number MEC-2021-0248. The study was conducted in accordance with the Declaration of Helsinki. Written informed consent was obtained from caregivers.

### Laboratory measurements

Hb and HbF levels were determined using the ABL90 FLEX + blood gas analyser (Radiometer, Denmark). Samples were obtained from arterial, capillary, or venous punctures performed as part of routine clinical care; no blood was drawn specifically for study purposes. HbF results were reported as the fraction of total Hb concentration.

### Statistical analysis

Statistical data analyses were conducted in R (version 4.5.1; R Foundation for Statistical Computing, Vienna, Austria) using dplyr for data management, ggplot2 for visualization, lme4 for mixed-effects modelling, and survival for time-to-event analyses.

The primary aim of this study was to characterize gestational age-specific HbF values and longitudinal HbF trajectories using the most complete dataset available. Therefore, all neonates meeting the predefined inclusion and exclusion criteria during the study period were included. As the study was designed to include the complete eligible cohort rather than a sampled population, a formal a priori sample size calculation was not indicated. The primary and secondary outcomes and corresponding analyses were described in the study protocol (Appendix A).

We used descriptive statistics to summarize clinical and laboratory characteristics, as well as impact of transfusion on study outcomes. Normality was assessed using the Shapiro–Wilk test in combination with visual inspection of histograms and Q–Q plots. In case of a normal distribution, data are presented as mean and standard deviation (SD). In case of a skewed distribution, as median and interquartile range (IQR). Categorical data are presented as numbers with percentages. No missing data were present for the variables included in the analyses. A p-value of <0.05 was considered significant. For GA-specific reference values, HbF measurements on day 0 were plotted against GA (per week and day) with LOESS (locally estimated scatterplot smoothing) and 95% confidence intervals. Postnatal HbF trajectories were visualized by postnatal age and stratified by GA subgroup (24–25, 26–27, 28–29 weeks) using LOESS curves with 95% confidence intervals (95% CI). For descriptive visualization, LOESS smoothing was implemented using the default smoothing span of 0.75 and a quadratic local polynomial. These parameters were used to provide a smooth representation of the observed trends. The LOESS curves were not used for statistical inference.

Time to first transfusion was analysed with Kaplan–Meier survival curves and compared between GA groups using the log-rank test.

The association between RBC transfusion exposure and HbF decline was assessed using linear mixed-effects models with repeated HbF measurements as the dependent variable. Postnatal age was selected as the primary time scale because the aim was to describe HbF trend of trajectory after birth, when postnatal haematopoiesis and RBC transfusion exposure begin. GA group was included as a covariate to account for differences in maturity at birth. All available HbF measurements were included in the longitudinal analyses; no averaging, down-sampling, or selection of closely spaced measurements was performed, as linear mixed effect models can adequately adjust for repeated measures. Postnatal age was modelled using natural cubic splines to estimate the non-linear association between postnatal age and HbF. Fixed effects included postnatal age, transfusion category (0, 1–3, ≥4), GA group, and total Hb concentration. An interaction between age and transfusion category was included to evaluate whether transfusion exposure modified the HbF trajectory. A random effect per patient accounted for repeated measurements.

To explore differences in HbF trajectories according to prematurity-related disease status, similar mixed-effects models were used for any prematurity-related disease, ROP, and BPD separately. The same spline specification and random-effects structure were applied in the outcome models. These models included postnatal age, disease status, gestational age group, and total haemoglobin concentration as fixed effects, with an interaction between postnatal age and disease status to compare HbF trajectories between neonates with and without the respective outcome.

Additional analyses performed in response to peer review were considered post-hoc and included the analysis of absolute HbF concentrations and HbF trajectories according to survival status. No sensitivity analyses were performed.

### Role of the funding source

No funding was received for this work.

## Results

A total of 856 neonates were included within the day-0 reference cohort (Fig. 1), with median GA of 31 weeks (IQR:28 0/7–35 5/7), median birth weight of 1432.5 g (IQR 1010–2505). Of these, 411/856 (48%) were males. Baseline characteristics are shown in Table 1.

**Fig. 1:**
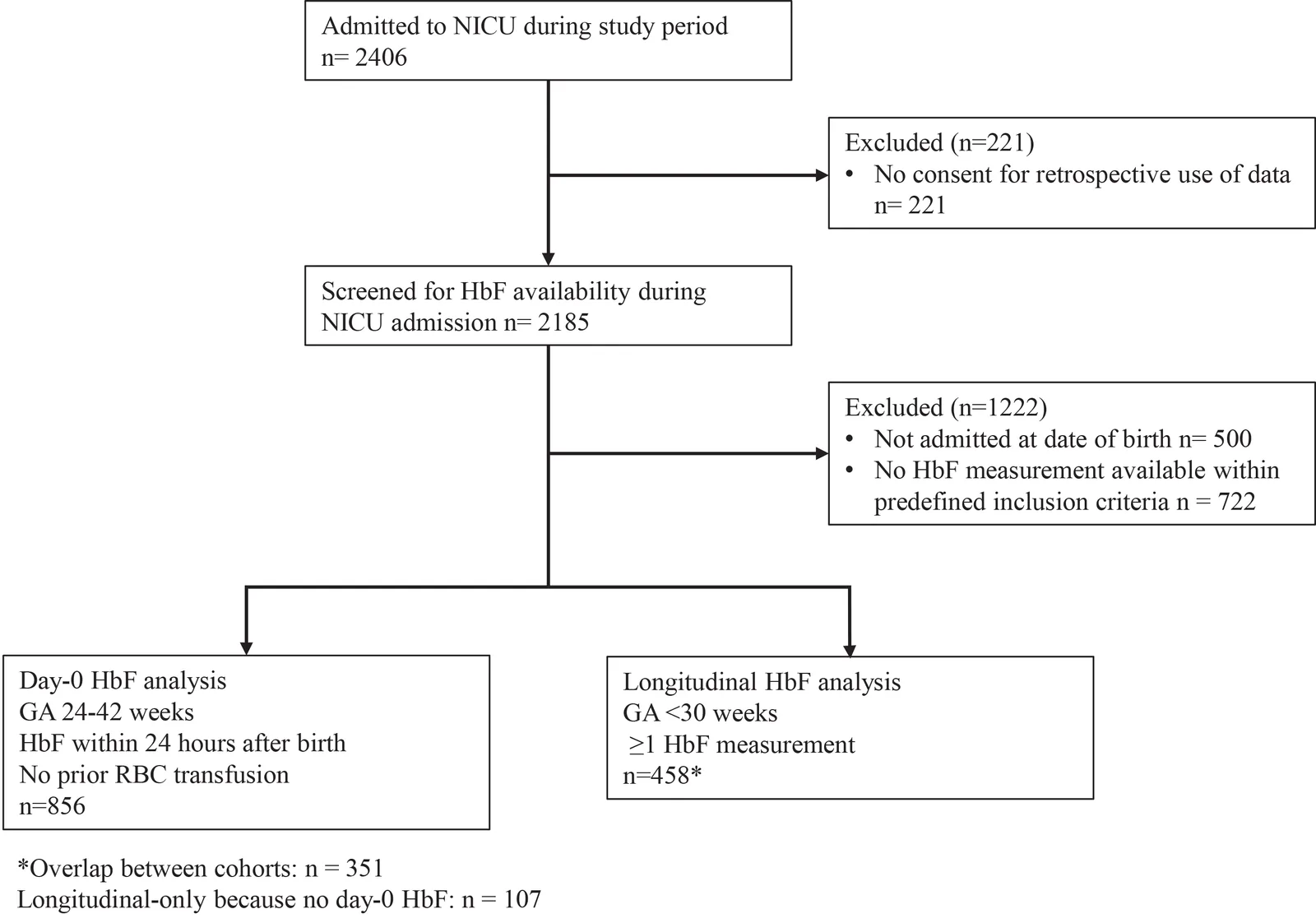
Study profile. **Legend:** Flowchart showing participant selection for the day-0 HbF reference analysis and the longitudinal HbF trajectory analysis. The two cohorts overlapped for 351 neonates. **Abbreviations:** HbF = fetal haemoglobin; GA = gestational age; NICU = neonatal intensive care unit; RBC = red blood cell.

**Table 1:** Patient characteristics.

Patient characteristics	Day 0 reference cohort, n = 856	<30 week GA longitudinal cohort, n = 458
Gestational age (weeks)–median (IQR)	31 0/7 (28 0/7–35 5/7)	27 5/7 (26 0/7–28 6/7)
Birth weight (grams)–median (IQR)	1432.5 (1009–2505)	987.5 (785–1200)
Male sex—n (%)	411 (48)	262 (57)
Female sex—n (%)	445 (52)	196 (43)
Singleton birth—n (%)	712 (83)	370 (81)
Caesarean section—n (%)	464 (54)	303 (66)
Apgar score at 5 min–median (IQR)	8 (7–9)	8 (7–9)
Duration of NICU stay (days)–median (IQR)	18 (8–47)	29.5 (13–60)
HbF on day of birth (%)—median (IQR)	88 (82–93)	89 (84–94)^a^
Hb on day of birth (mmol/L)—median (IQR)	8.7 (7.4–10.3)	8.7 (7.6–10.1)^a^

Abbreviations: Hb = haemoglobin; HbF = fetal haemoglobin; n = number, NICU = neonatal intensive care unit; RBC = red blood cell; IQR = interquartile range.

a Available for 351 patients.

Median HbF values at birth remained ≥0.85 and with a small variability (IQR: 81–92) for neonates born between 24 and 35 weeks, as shown in Fig. 2. Between week 35 and 42, HbF gradually declined, reaching median values of 0.74 at term (40 weeks), but with a wider variability between individual neonates (IQR 0.70–0.83).

**Fig. 2:**
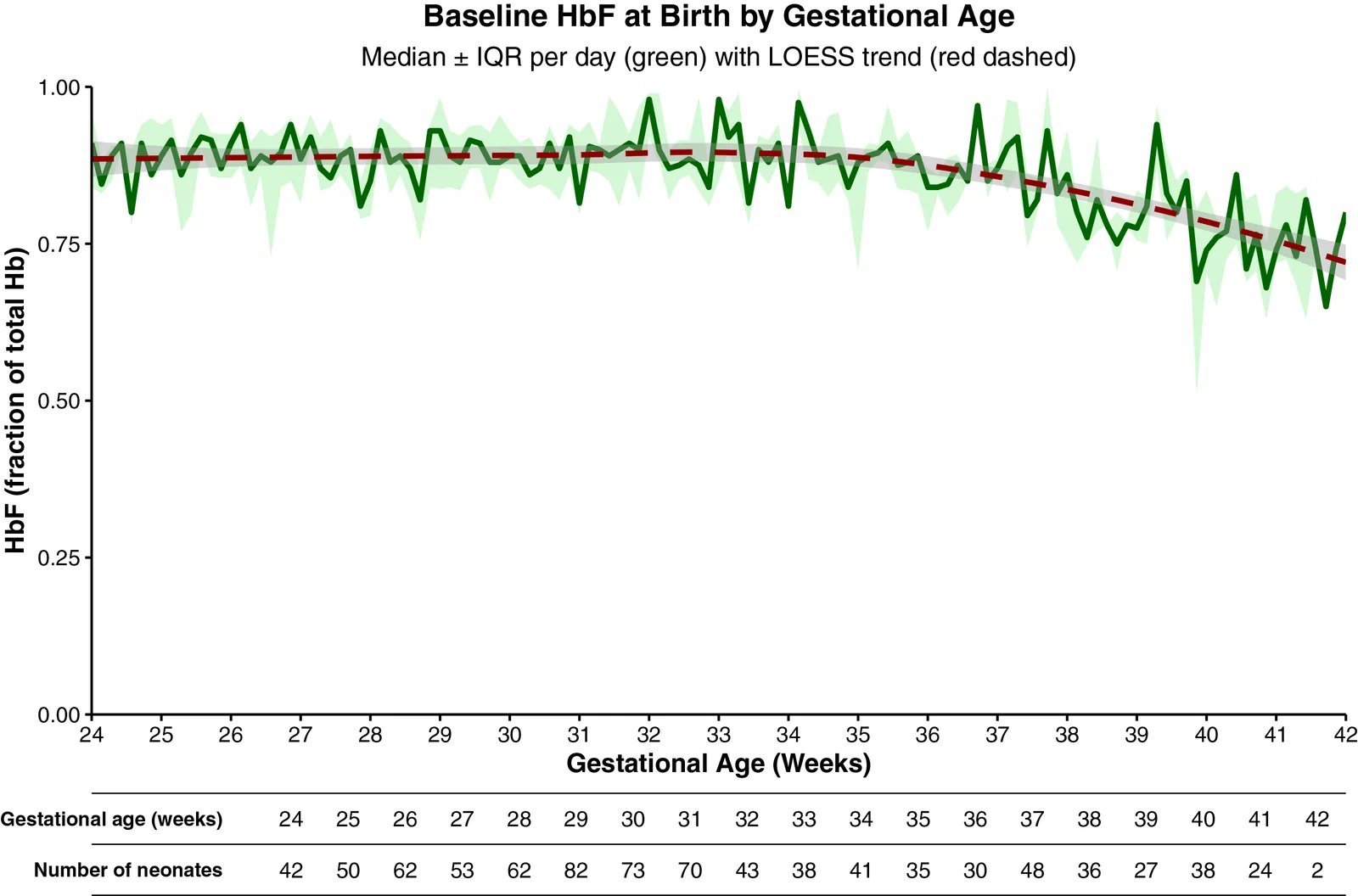
Baseline HbF at birth by gestational age. **Legend:** Median ± IQR per GA day (green) with LOESS smoothing (red dashed line) and 95% confidence interval (shaded area). Numbers below the x-axis indicate the number of unique neonates contributing to the day-0 HbF analysis within each completed gestational week. **Abbreviations:** HbF = fetal haemoglobin; GA = gestational age.

The longitudinal cohort consisted of 458 neonates born before 30 weeks of GA. (See Table 1, Fig. 1). In this very preterm group 9461 paired Hb and HbF measurements were available for longitudinal analysis with a median of 11 HbF measurements per infant (IQR 5–25). Median HbF at birth was 0.87 (IQR: 0.81–0.92), with a median total Hb concentration of 8.7 mmol/L (IQR: 7.6–10.1).

As shown in Fig. 3, HbF declined during the first 40 postnatal days across all GA groups, with the steepest decline observed in the neonates born at 24–25 weeks' GA. In neonates born between 24 and 27 weeks’ GA, a spontaneous HbF increase occurred after day 40, with a median rise from the nadir of 7.0% (IQR 0.0–18.0). However, this rebound did not restore HbF to the levels observed in the 28–29 weeks group at the same time point (median 50.0%, IQR 27.3–57.5).

**Fig. 3:**
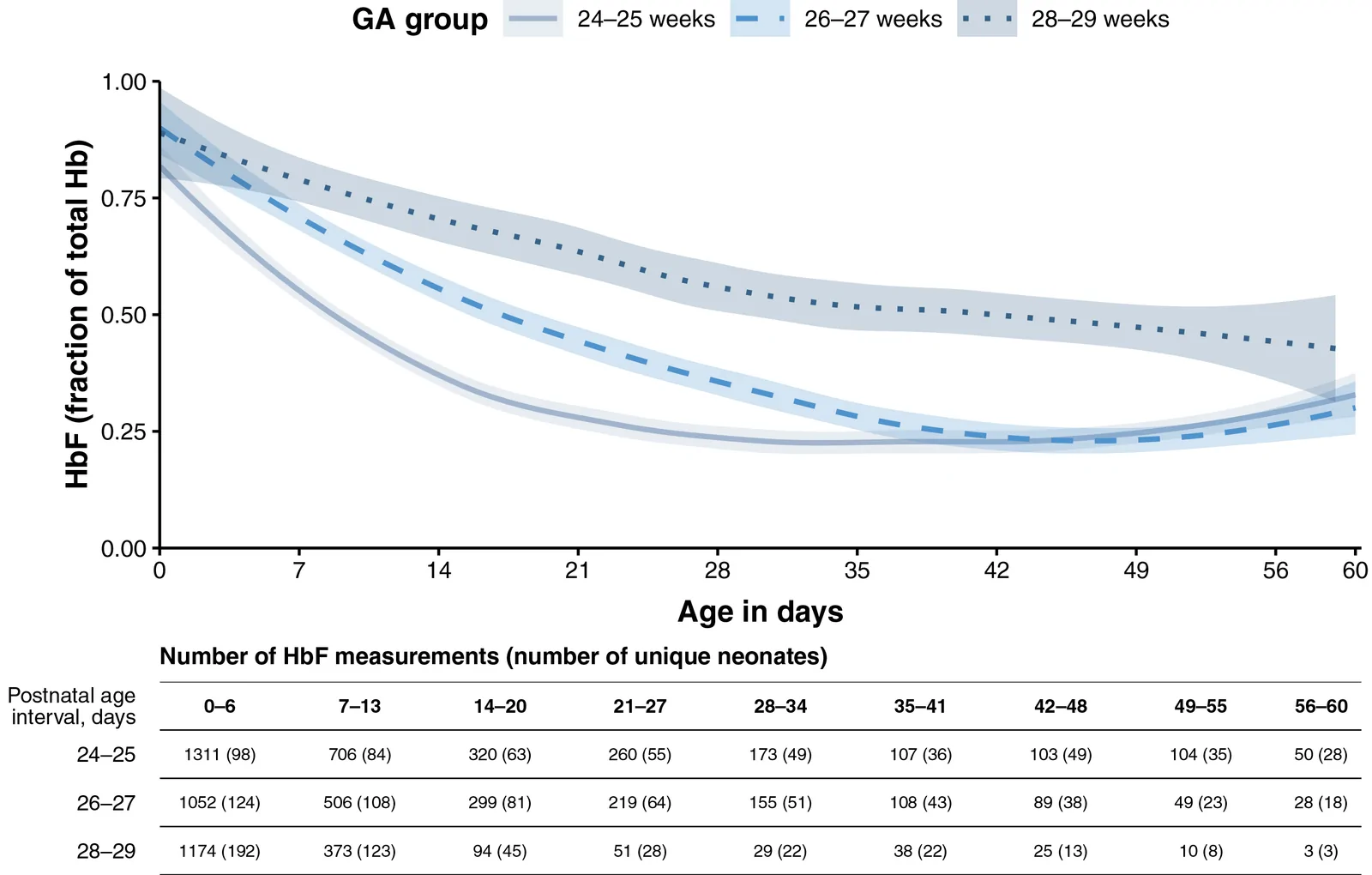
Median HbF by postnatal age for neonates born between 24 and 30 weeks GA (n = 458). **Legend:** LOESS smoothing curve with 95% CI. Numbers below the figure indicate the number of HbF measurements, with the number of unique neonates shown in parentheses, contributing within each seven-day postnatal age interval and GA group. **Abbreviations:** HbF = fetal haemoglobin; GA = gestational age.

Of the 458 neonates born before 30 weeks’ gestation, 227/458 (50%) received at least one RBC transfusion during admission. In total 656 RBC transfusions were given, resulting in a mean of 1.4 RBC transfusions per patient (SD 1.9, range: 0–6). See Table 2. The highest transfusion frequency occurred in neonates born between 24 and 25 weeks, with a median of three RBC transfusions (IQR: 1–5), compared to one (IQR: 0–3) in the 26–27 weeks group and zero (IQR: 0–2) in the 28–29 weeks group. The proportion of neonates receiving at least one transfusion decreased with increasing GA, from 102/111 (92%) in those born at 24–25 weeks to 74/139 (53%) at 26–27 weeks and 51/208 (25%) at 28–29 weeks. This resulted in a significantly lower transfusion-free survival in the subgroup 24–25 weeks of GA (log-rank p < 0.0001, see Fig. 4).

**Table 2:** Specific parameters related to outcome, specific for <30 week GA longitudinal cohort n = 458.

Patients that received RBC transfusion—n (%)	227 (50)
Total number of RBC transfusions given—n	656
Mean RBC transfusions per patient∗ (range)	1.4 per patient (0–6)
Severe prematurity related outcomes—n (%)	203 (44)
ROP—n (%)	51 (11)
BPD—n (%)	121 (26)
NEC—n (%)	26 (6)
Death—n (%)	69 (15)

Abbreviations: RBC = red blood cell; IQR = interquartile range; ROP = retinopathy of prematurity; BPD = bronchopulmonary dysplasia; NEC = necrotizing enterocolitis.

**Fig. 4:**
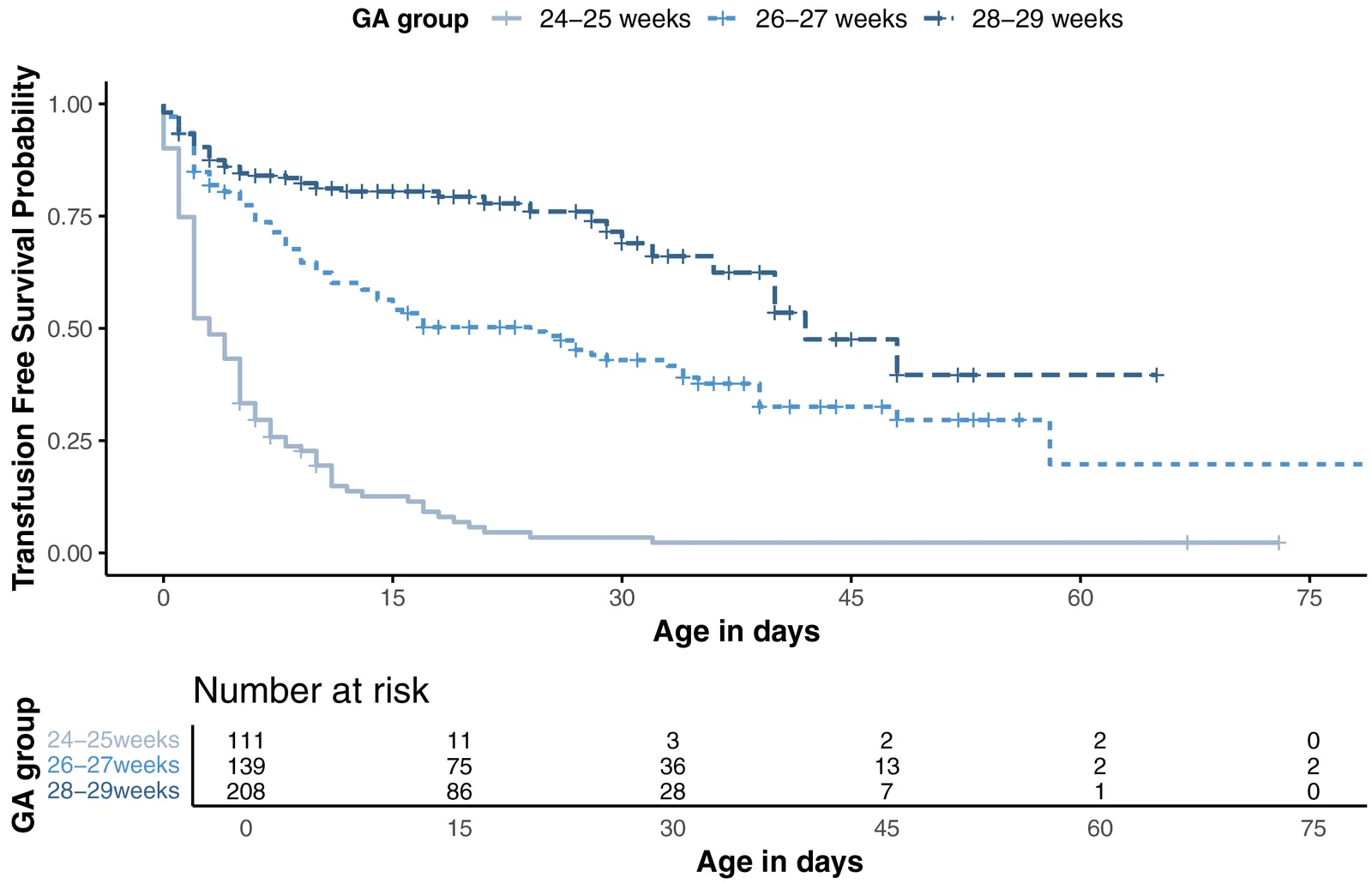
Time to first red blood cell transfusion per gestational age group. **Legend:** Kaplan–Meier survival analysis of transfusion-free survival. **Abbreviations:** RBC = red blood cell; GA = gestational age.

The association between RBC transfusions and postnatal HbF course is illustrated in Fig. 5. Among neonates who did not receive any RBC transfusions, median HbF levels declined gradually during the first weeks of life but remained relatively high, with adjusted HbF values consistently above 0.75. In contrast, neonates who received RBC transfusions showed a markedly sharper and earlier decline in HbF, which was most pronounced in those receiving four or more transfusions, reaching nadir levels of approximately 0.17. Following this transfusion-associated decline, HbF levels increased modestly; however, they remained substantially below those observed in neonates who did not receive RBC transfusions. At the end of follow-up, adjusted HbF levels were approximately 0.28 and 0.50 lower in the 1–3 and ≥4 transfusion groups, respectively, compared with neonates who did not receive RBC transfusions. These patterns were independent of GA group and Hb concentration at sampling, and the interaction between postnatal age and transfusion group was statistically significant (p < 0.001, Supplementary Table S1).

**Fig. 5:**
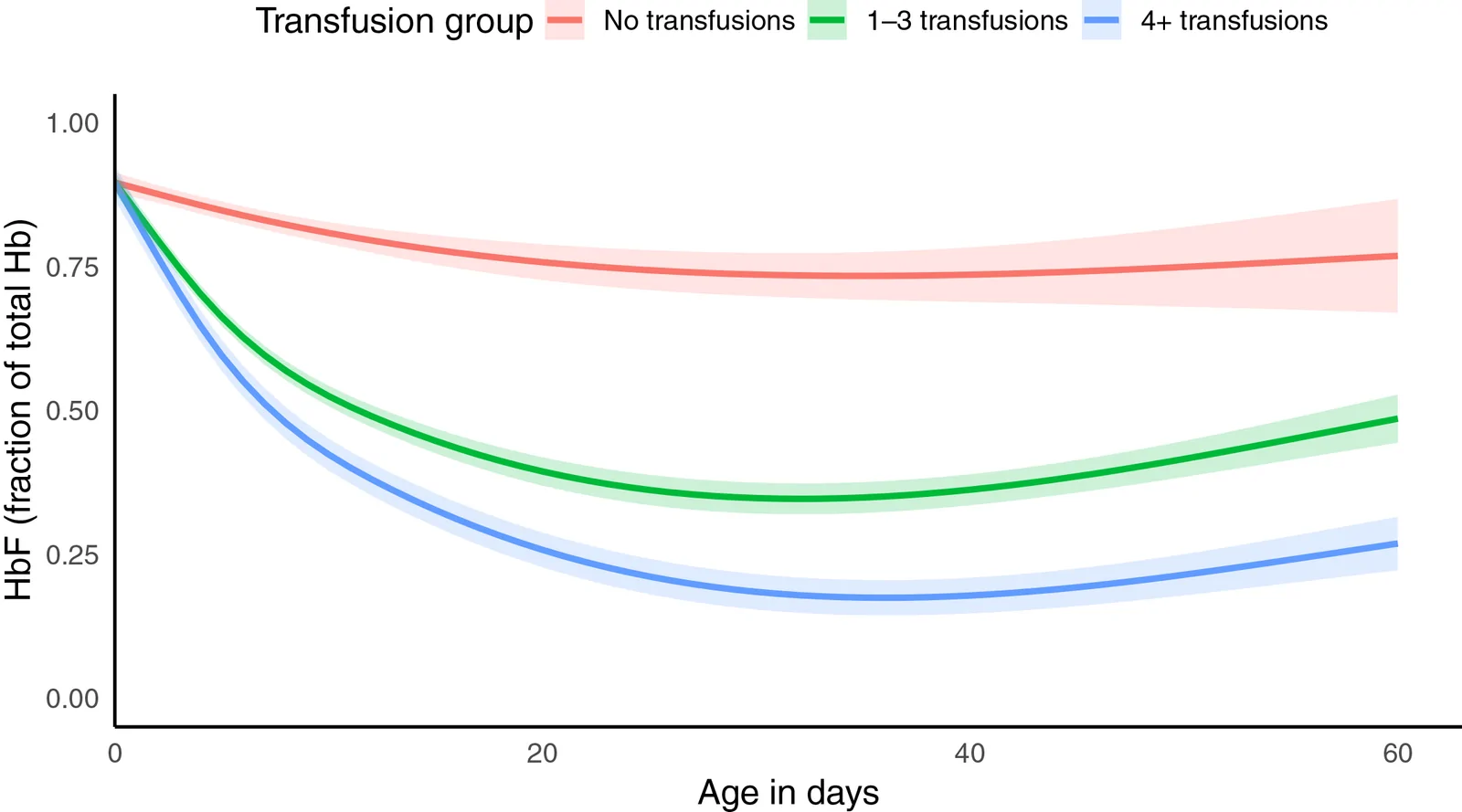
Modelled postnatal HbF trajectories by transfusion exposure category. **Legend:** Curves stratified by 0, 1–3, or ≥4 transfusions, adjusted for GA group and Hb concentration. The interaction between postnatal age and transfusion exposure was statistically significant. **Abbreviations:** Hb = haemoglobin; HbF = fetal haemoglobin; GA = gestational age.

In the <30-week group (n = 458), 203/458 (44%) neonates developed at least one severe prematurity-related disease (composite outcome: severe ROP, BPD, NEC and/or death before discharge; see Table 2). Individual outcomes were not mutually exclusive and could occur in the same patient, the overlap between the individual prematurity-related outcomes is shown in Supplementary Table S2.

In linear mixed-effects models including spline terms for postnatal age, baseline HbF levels at birth did not significantly differ between neonates with and without the composite outcome. However, over time, neonates who developed severe ROP, BPD, NEC, or died exhibited a significantly steeper postnatal decline in HbF compared with those without major morbidity or mortality (overall interaction between outcome and postnatal age, p < 0.001; Fig. 6A). When individual outcomes were evaluated separately, similar patterns were observed. Neonates requiring treatment for ROP demonstrated a markedly steeper HbF decline compared with those without treated ROP (p < 0.001; Fig. 6B). Likewise, a significantly steeper HbF decline was observed in neonates who subsequently developed severe BPD (p < 0.001; Fig. 6C, Supplementary Table S1).

**Fig. 6:**
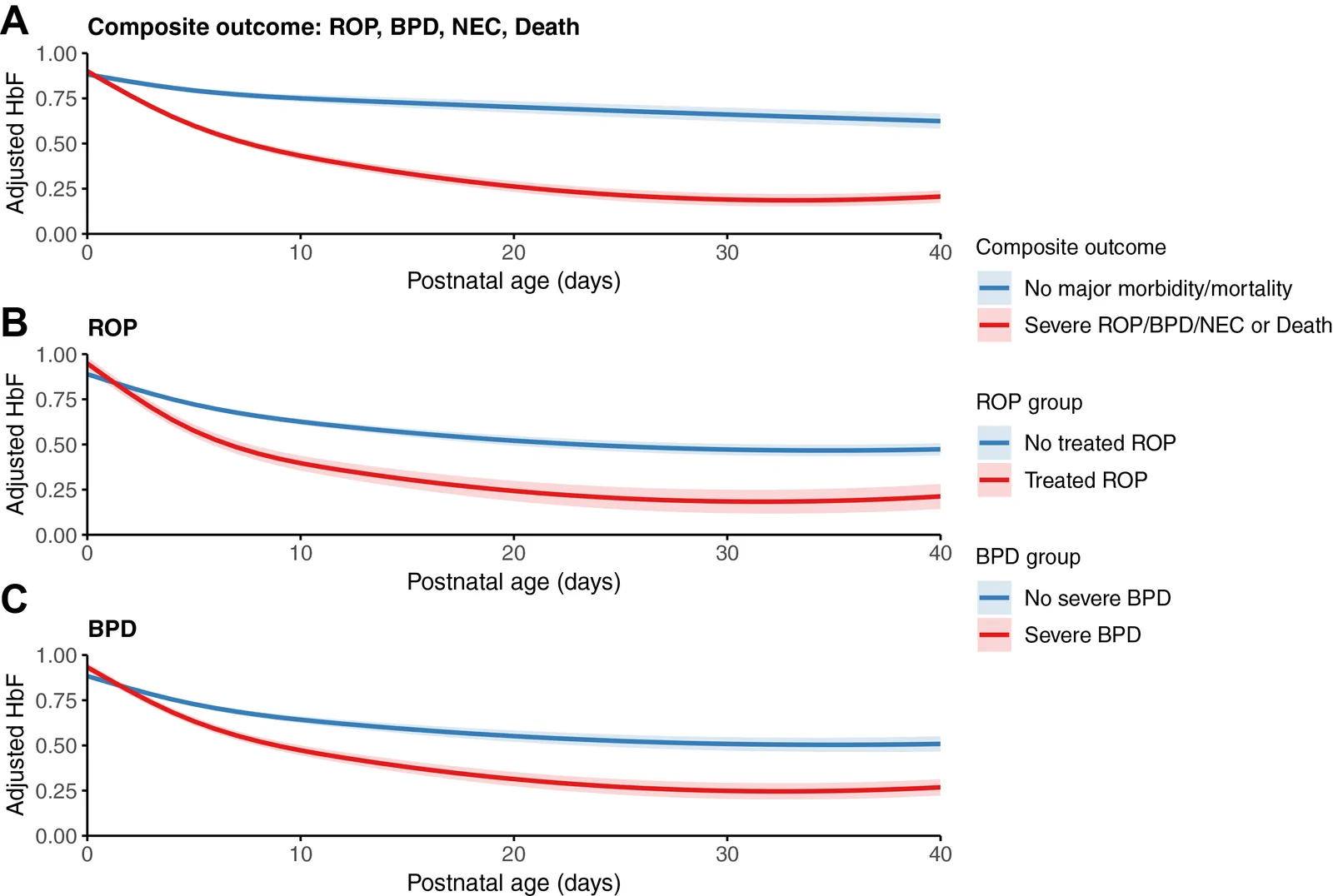
Adjusted fetal haemoglobin trajectories by major prematurity-related outcomes. **Legend:** Adjusted fetal haemoglobin (HbF) trajectories over the first 40 postnatal days stratified by major prematurity-related outcomes. Panels show (A) composite outcome (severe ROP, severe BPD, NEC, or death), (B) ROP requiring treatment, and (C) severe BPD. Lines represent model-based predicted HbF values from mixed-effects spline models adjusted for gestational age group and haemoglobin concentration. Shaded areas indicate 95% confidence intervals. The composite outcome analysis included 203 neonates with severe prematurity-related morbidity or death (5558 HbF measurements) and 255 neonates without the composite outcome (2509 HbF measurements). The treated ROP analysis included 51 neonates with treated ROP (1931 HbF measurements) and 407 neonates without treated ROP (6163 HbF measurements). The severe BPD analysis included 121 neonates with severe BPD (3689 HbF measurements) and 337 neonates without severe BPD (4378 HbF measurements). **Abbreviations:** HbF = fetal haemoglobin; ROP = retinopathy of prematurity; BPD = bronchopulmonary dysplasia; NEC = necrotizing enterocolitis.

## Discussion

We demonstrated that GA–specific reference values for HbF at birth are relatively high in neonates born between 24 and 35 weeks of gestation and are comparable across this range. From approximately 35 weeks of gestation onward, median HbF levels at birth are lower. This is consistent with the physiological Hb switch from fetal to adult Hb that begins in late gestation and is completed during the first 6–12 months of postmenstrual age. In addition, we confirmed that HbF declines steeply during the first postnatal weeks in neonates born before 30 weeks’ gestation.^3^^,^^4^^,^^11^ This decline was most pronounced in neonates born at 24–25 weeks of gestation and was already evident after a single RBC transfusion. Although a modest spontaneous increase in HbF was observed after approximately postnatal day 40 in neonates born between 24 and 27 weeks of gestation, HbF levels did not recover to those observed in more mature neonates, suggesting limited compensatory capacity of endogenous fetal erythropoiesis.

Furthermore, we demonstrated that RBC transfusions were given most frequently to neonates born at 24–25 weeks, contributing to the steepest HbF decline in the same age-group. This relation to RBC transfusion and provoked HbF decline has previously been reported.^3^ In our cohort, longitudinal modelling of HbF trajectories in relation to transfusion exposure demonstrated that RBC transfusions were associated with an accelerated decline in HbF over time compared with neonates who did not receive RBC transfusions. Transfusion exposure was categorized according to the total number of RBC transfusions received during follow-up rather than modelled as a time-varying exposure. This approach enabled comparison of HbF trajectories across clinically meaningful levels of overall transfusion burden while maintaining a model that was readily interpretable. Although modelling transfusion exposure as a continuously updated time-varying variable may provide a more biologically representative assessment of the temporal relationship between RBC transfusions and HbF trajectories, it would substantially increase model complexity and reduce interpretability. The interval between successive transfusions was not analysed separately; therefore, our model does not distinguish between similar cumulative transfusion exposure occurring over shorter versus longer time intervals, which may differentially affect HbF decline and recovery. Future studies incorporating time-varying cumulative transfusion exposure or analyses centred on individual transfusion events may further refine the temporal dynamics of HbF decline and recovery. The observed differences in transfusion-free survival between GA groups further support the interpretation of the observed relationship between transfusion exposure and HbF trajectories.^19^ These findings underscore the importance of formerly implemented restrictive transfusion triggers in the very preterm neonates,^20^ but also provide ground to further explore novel strategies to preserve endogenous HbF levels. Despite the new transfusion guideline, RBC transfusions are still indicated as severe anaemia in preterm neonates is negatively associated with impaired cognitive outcome.^21^

One such novel HbF-preserving approach currently investigated, is the use of umbilical cord blood–derived RBC products from placentas from term, healthy neonates.^22^^,^^23^ Recently, the randomized BORN trial demonstrated that cord blood-derived RBC transfusions successfully preserved circulating HbF levels in extremely preterm neonates and reported encouraging clinical findings, including reduced severity of ROP and BPD in the per-protocol analysis.^24^ Although larger randomized studies are needed to confirm clinical efficacy, these findings support the feasibility and physiological rationale of HbF-preserving transfusion strategies. In this context, our observational study complements the BORN trial by providing GA-specific reference values and longitudinal HbF trajectories that can serve as a physiological benchmark for the development and evaluation of HbF-preserving transfusion strategies. These data may also aid in defining transfusion targets for novel, cord-blood derived RBCs as the HbF levels associated with optimal clinical outcomes remain unknown.

Lastly, we observed that neonates who subsequently developed the composite outcome of severe prematurity related disease and mortality exhibited a steeper postnatal decline in HbF than those who did not. This association was most pronounced for treated ROP and was also evident for severe BPD, whereas no consistent association was observed for NEC in our cohort. The composite outcome was intended to capture the overall burden of severe prematurity-related morbidity and mortality, while the individual analyses indicate that the overall signal was most pronounced for ROP and BPD. When analysed separately, neonates who died before discharge also exhibited a steeper HbF decline than neonates who survived to discharge (Supplementary Figure S1; Supplementary Table S1). However, these infants died at a median postnatal age of 7 days (IQR 5–15), resulting in substantially shorter longitudinal follow-up. Consequently, the estimated HbF trajectory for this group was based predominantly on early postnatal measurements and should therefore be interpreted with caution.

Although RBC transfusions substantially influence HbF trajectories, transfusion exposure alone did not fully account for the observed associations with ROP and BPD. As outcome status was used to retrospectively define the comparison groups, these findings should be interpreted as descriptive associations rather than predictive or causal relationships. Similarly, infants requiring greater clinical support generally underwent blood gas analyses more frequently and therefore contributed a higher number of longitudinal HbF measurements than infants with less severe illness. Furthermore, residual confounding by illness severity and other clinical factors including respiratory support, oxygen exposure, infection, PDA treatment, and blood loss cannot be excluded. Together, these findings suggest that HbF decline may reflect a broader vulnerability profile rather than a single causal pathway, and that multiple interacting clinical and developmental factors likely contribute to adverse outcomes in extremely preterm neonates.

Because HbF has a higher oxygen affinity than HbA, higher HbF levels may limit excessive tissue oxygenation during early postnatal life. Rapid replacement of HbF by adult donor RBCs following transfusion has been proposed to increase oxygen exposure in vulnerable tissues such as the immature lung and retina, potentially contributing to oxidative stress–related injury.^11^ Although oxygenation parameters were not assessed directly, the lower HbF trajectories observed in neonates who developed prematurity-related diseases are consistent with this conceptual framework.

Importantly, HbF trajectories are likely shaped by a complex interplay of factors beyond transfusion exposure, including GA, intrauterine growth restriction, perinatal hypoxia, infection, inflammation, and clinical interventions.^25^, ^26^, ^27^, ^28^ Transfusions may suppress endogenous erythropoiesis through feedback inhibition, but within a broader physiologic and pathologic context.^29^ In addition, fetal RBCs differ from adult donor cells not only in HbF content but also in size, deformability, membrane composition, and resistance to oxidative stress, all of which influence oxygen delivery and tissue vulnerability.^6^ Together, these mechanisms may contribute to the physiologic advantages of maintaining a more fetal-like red cell profile in extremely preterm neonates. However, these factors were not specifically evaluated in the present study, and their independent contribution to HbF decline remains to be established.

Our study has several strengths and limitations. A major strength of this study is the use of longitudinal modelling based on >9000 paired Hb and HbF measurements, which allowed us to characterize individual HbF trajectories and their association with transfusion exposure with greater resolution than single time-point analyses. This approach provided a more nuanced understanding of how transfusion timing and frequency shape Hb composition over time. The large sample size, inclusion of multiple GA groups, and use of routinely collected blood gas data strengthen the robustness and clinical relevance of our findings. As HbF measurements were obtained routinely as part of blood gas analyses during clinical care rather than for specific clinical indications, we consider the included cohort to be representative of the tertiary NICU population at our institution during the study period. Nevertheless, our findings should be interpreted within the context of a single-centre tertiary NICU and are not directly generalizable to healthy term neonates or neonatal populations with different case-mix or transfusion practices.

Postnatal age was used as the primary time scale to characterise changes in circulating HbF following preterm birth, given the relevance of circulating HbF to oxygen-release characteristics during this period. GA group was included to account for differences in maturity at birth. However, the physiological haemoglobin switch is intrinsically related to fetal maturation and postmenstrual age; our findings should therefore not be interpreted as describing the developmental haemoglobin switch according to postmenstrual age.

A limitation in our study is the fact that the HbF fraction was measured on the ABL90 FLEX + blood gas analyser. In general, the gold standard for HbF quantification is through high-performance liquid chromatography. Previous validation studies have demonstrated a systematic positive bias of hemoximeter-based HbF measurements compared with HPLC, suggesting that HbF values in our cohort may be slightly overestimated.^30^ In line with broader blood gas analyser studies, hemoximetric hemoglobin measurements typically show a small systematic deviation of approximately 2–5% compared with laboratory reference methods, with 95% limits of agreement in the range of ±1.5–2 g/dL at the individual measurement level.^31^ However, as our analyses primarily focused on longitudinal changes rather than absolute values at a single time point, this limited systematic bias is unlikely to materially affect the interpretation of HbF trajectories. Moreover, the larger blood volume required for HPLC-based measurements limits its feasibility in very preterm neonates, supporting the use of blood gas–derived measurements in this population. Finally, the study period overlapped with the COVID-19 pandemic. Although we are not aware of changes in local transfusion practices, HbF measurement protocols, or neonatal management that would be expected to systematically affect HbF trajectories, indirect effects of the pandemic on healthcare delivery cannot be completely excluded.

In conclusion, we demonstrate that HbF is the dominant Hb fraction at birth in neonates born between 24 and 42 weeks of gestation and is comparable at levels above 70% until approximately 35 weeks of gestation. From late gestation onward, toward term, HbF levels at birth are lower, providing GA–specific reference values for both preterm and term neonates. In very preterm neonates born before 30 weeks of gestation, HbF declines rapidly during the early postnatal period, with the steepest decrease observed in neonates born at 24–25 weeks’ gestation. This decline is strongly associated with exposure to adult-derived RBC transfusions, which substantially accelerate the replacement of circulating HbF by HbA. Furthermore, a steeper, transfusion-associated decline in HbF was significantly correlated with the development of prematurity-related diseases, most notably ROP and BPD. Although preserving fetal haematopoiesis during early postnatal life in neonates born before 28 weeks of gestation may reduce the risk of these complications, transfusion exposure alone did not fully explain all observed morbidity, indicating that additional risk factors are involved. These observational findings provide physiological evidence supporting the hypothesis that preservation of circulating HbF may be relevant to prematurity-related outcomes, although causal relationships remain to be established. Taken together, these findings support further evaluation of HbF-preserving strategies, including novel transfusion approaches, while emphasizing the need for continued investigation into other determinants of adverse outcomes in extremely preterm neonates.

## Contributors

ANT, NHGP and JHD designed the study. JHD and ANT collected the data. JHD and NHGP accessed, verified and analysed the data and drafted the manuscript. TRLK, HRT, EJH, PMS, IKMR and EL revised the manuscript critically for important intellectual content. All authors approved the final version of the manuscript. JHD is the guarantor.

## Data sharing statement

Deidentified individual participant data underlying the results reported in this Article, together with a data dictionary, can be made available upon reasonable request. Data will be available from the time of publication until 5 years thereafter. Requests should be directed to the corresponding author. Data will be shared with researchers who provide a methodologically sound proposal, subject to approval by the study investigators and in accordance with institutional and ethical regulations.

## Declaration of interests

We declare no competing interests.
