# Supplementary material for: Gestational age references and trajectory of fetal haemoglobin in relation to outcome in neonates born before 30 weeks of gestation: a single-centre retrospective cohort study in The Netherlands
Source: eClinicalMedicine. 2026 Oct 2;100:104256. doi: 10.1016/j.eclinm.2026.104256 (PMC13659609; doi:10.1016/j.eclinm.2026.104256)
Supplement: Supplementary file 1 [file mmc1.pdf]

SUPPLEMENTARY MATERIALS

Gestational age references and trajectory of fetal haemoglobin in relation to outcome in neonates born before 30 weeks of gestation: a single-centre retrospective cohort study in The Netherlands

Index

Supplementary Table S1 2
Supplementary Table S2 3
Supplementary Figure S1 4
Supplementary Figure S2 5

Supplementary tables

Supplementary Table S1. Fixed effects from linear mixed-effects models of postnatal HbF trajectories
Model specification: primary exposure effects and their interactions with postnatal age are shown. All models were adjusted for gestational age group and total haemoglobin concentration. Postnatal age was modelled using natural cubic splines. β = regression coefficient; CI = confidence interval.

**Panel A. HbF trajectory and transfusion exposure**			
**Predictor**	**β**	**95% CI**	**p-value**
**Transfusion 1–3**	-0.04	-0.06 to -0.02	0.001
**Transfusion ≥4**	-0.06	-0.09 to -0.02	0.002
**Time 1 × Transfusion 1–3**	-0.35	-0.42 to -0.28	<0.001
**Time 2 × Transfusion 1–3**	-0.50	-0.57 to -0.43	<0.001
**Time 3 × Transfusion 1–3**	-0.17	-0.27 to -0.07	0.001
**Time 1 × Transfusion ≥4**	-0.47	-0.54 to -0.40	<0.001
**Time 2 × Transfusion ≥4**	-0.73	-0.81 to -0.66	<0.001
**Time 3 × Transfusion ≥4**	-0.33	-0.44 to -0.23	<0.001
**Panel B. HbF trajectory and composite outcome**			
**Predictor**	**β**	**95% CI**	**p-value**
**Composite outcome**	0.02	-0.01 to 0.04	0.192
**Time 1 × Composite outcome**	-1.05	-1.21 to -0.89	<0.001
**Time 2 × Composite outcome**	1.53	1.05 to 2.02	<0.001
**Time 3 × Composite outcome**	4.59	3.54 to 5.64	<0.001
**Panel C. HbF trajectory and treated ROP**			
**Predictor**	**β**	**95% CI**	**p-value**
**Treated ROP**	0.06	0.02 to 0.10	0.003
**Time 1 × Treated ROP**	-0.53	-0.72 to -0.33	<0.001
**Time 2 × Treated ROP**	0.20	-0.19 to 0.58	0.326
**Time 3 × Treated ROP**	1.51	0.63 to 2.39	<0.001
**Panel D. HbF trajectory and treated BPD**			
**Predictor**	**β**	**95% CI**	**p-value**
**Severe BPD**	0.05	0.02 to 0.08	<0.001
**Time 1 × Severe BPD**	-0.60	-0.77 to -0.43	<0.001
**Time 2 × Severe BPD**	0.35	-0.04 to 0.73	0.077
**Time 3 × Severe BPD**	1.67	0.80 to 2.54	<0.001
**Panel E. HbF trajectory and death before discharge**			
**Predictor**	**β**	**95% CI**	**p-value**
**Death before discharge**	-0.05	-0.08 to -0.02	0.003
**Time 1 × Death before discharge**	-1.03	-1.30 to -0.77	<0.001
**Time 2 × Death before discharge**	0.35	-0.32 to 1.02	0.305
**Time 3 × Death before discharge**	1.88	0.46 to 3.30	0.010

Supplementary Table S2. Overlap between individual prematurity-related outcomes in neonates born before 30 weeks' gestation

Outcome	ROP	BPD	NEC	Death
**ROP**	51	37	6	1
**BPD**	37	121	7	7
**NEC**	6	7	26	10
**Death**	1	7	10	69

*Death during NICU stay

Supplementary Figure S1. Postnatal HbF trajectories according to survival status. Adjusted postnatal HbF trajectories are shown for neonates who survived to discharge and those who died before discharge. Estimates were derived from linear mixed-effects models adjusted for gestational age group and total haemoglobin concentration, with postnatal age modelled using natural cubic splines. Shaded areas represent 95% confidence intervals. Neonates who died had a median postnatal age at death of 7 days (IQR 5–15); therefore, the estimated trajectory in this group is based predominantly on early postnatal measurements and should be interpreted with caution at later time points.

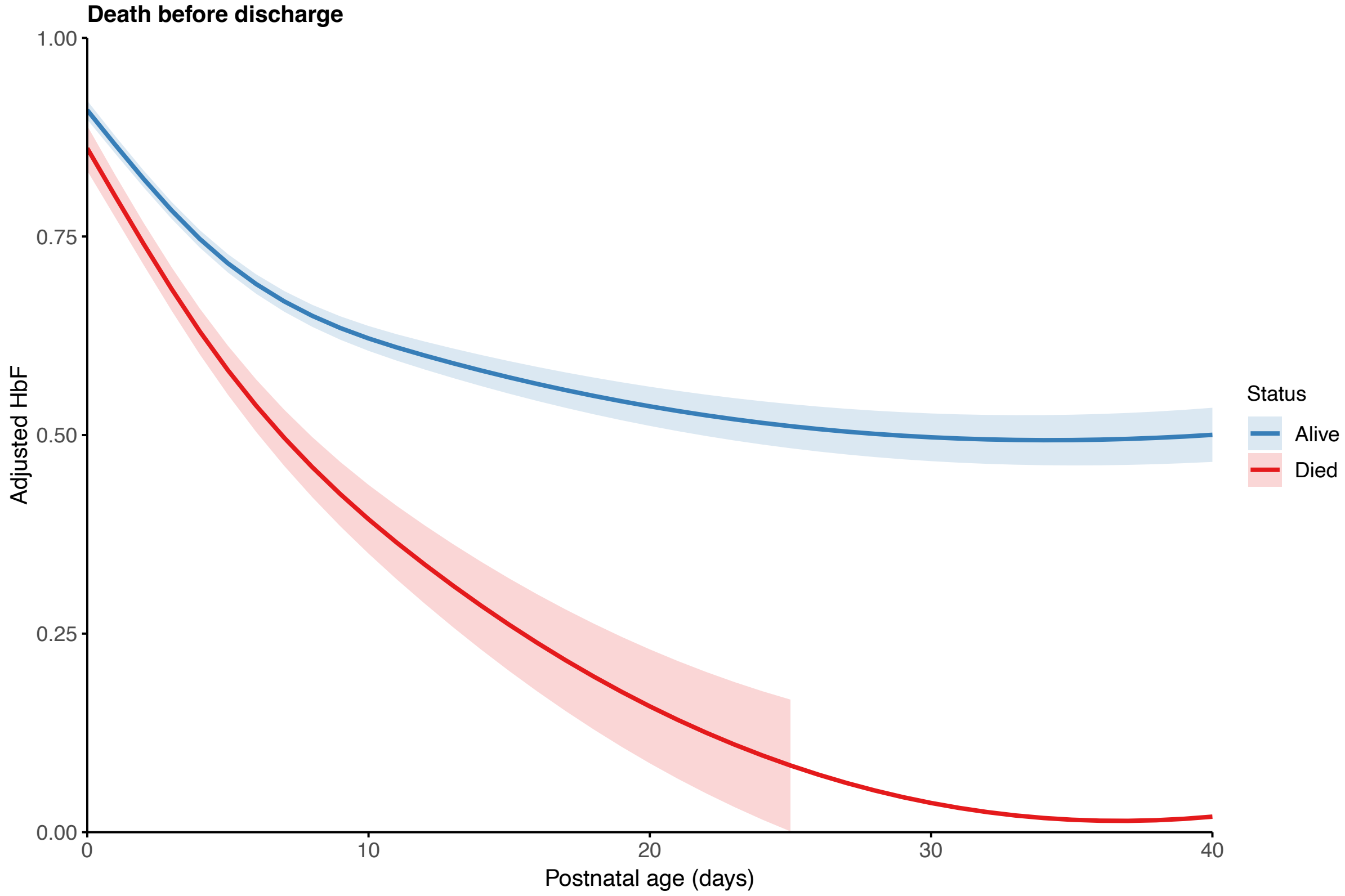

Supplementary Figure S2. Median absolute fetal haemoglobin by postnatal age in neonates born between 24 and 30 weeks of gestation. Absolute fetal haemoglobin (HbF) was calculated by multiplying the HbF fraction by the corresponding total haemoglobin concentration and is expressed in mmol/L. Lines represent LOESS-smoothed trajectories of the daily median absolute HbF concentration, stratified by gestational age group (24–25, 26–27, and 28–29 weeks). Shaded areas represent 95% confidence intervals. LOESS smoothing was performed with a span of 0.75.

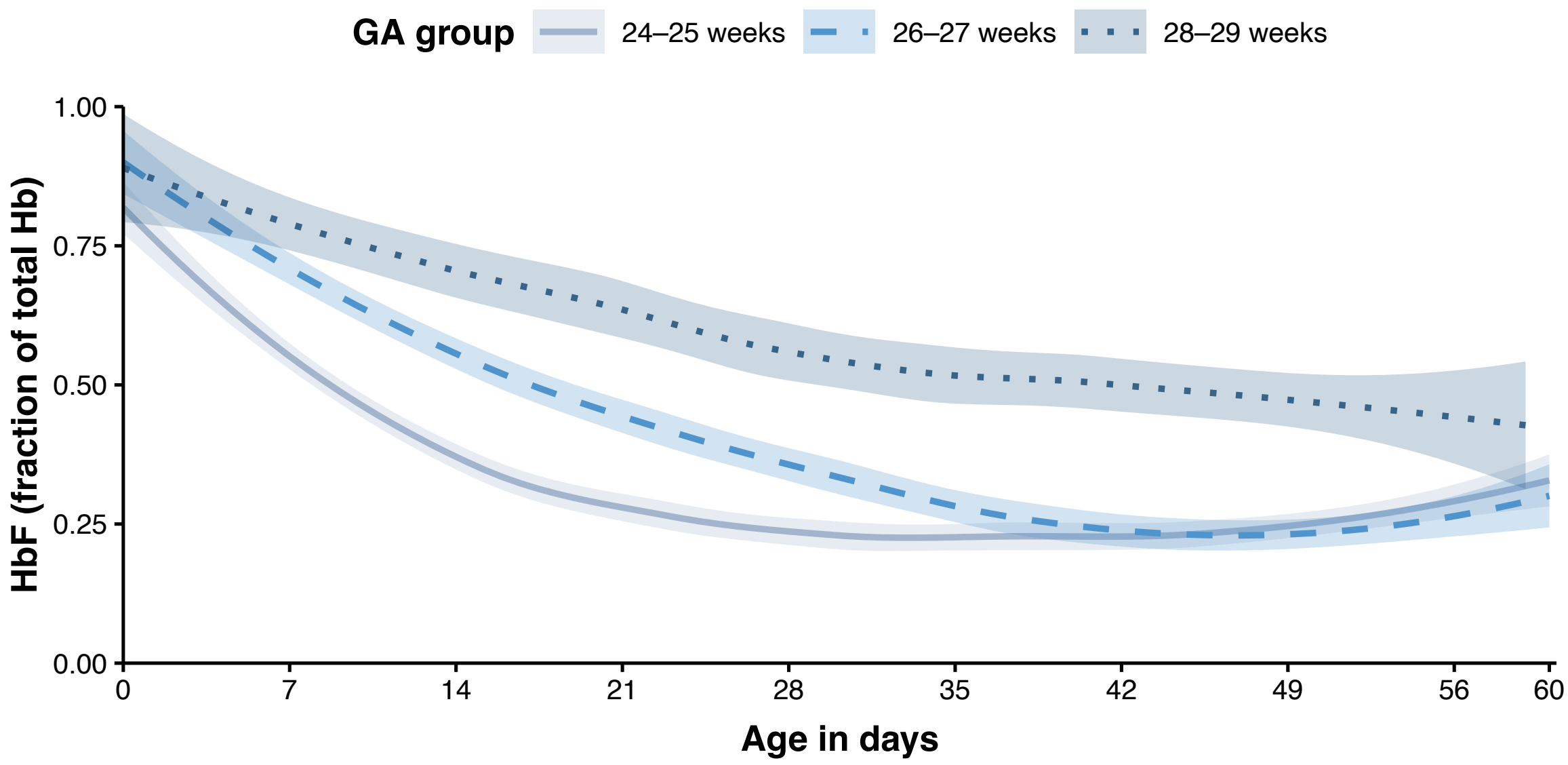

Number of HbF measurements (number of unique neonates)

Postnatal age interval, days	0–6	7–13	14–20	21–27	28–34	35–41	42–48	49–55	56–60
24–25	1311 (98)	706 (84)	320 (63)	260 (55)	173 (49)	107 (36)	103 (49)	104 (35)	50 (28)
26–27	1052 (124)	506 (108)	299 (81)	219 (64)	155 (51)	108 (43)	89 (38)	49 (23)	28 (18)
28–29	1174 (192)	373 (123)	94 (45)	51 (28)	29 (22)	38 (22)	25 (13)	10 (8)	3 (3)
