# Supplementary material for: Gestational age references and trajectory of fetal haemoglobin in relation to outcome in neonates born before 30 weeks of gestation: a single-centre retrospective cohort study in The Netherlands
Source: eClinicalMedicine. 2026 Oct 2;100:104256. doi: 10.1016/j.eclinm.2026.104256 (PMC13659609; doi:10.1016/j.eclinm.2026.104256)
Supplement: Supplementary file 2 [file mmc2.pdf]

TEMPLATE RESEARCH PROTOCOL
for non-WMO-applicable research

19-01-2023, versie 2

**Full title of protocol**	Fetal hemoglobin trajectory in extremely preterm neonates
**Short title or Acronym**	HbF-Tra
**Version**	1
**Date**	01/11/2024
**Principal investigator**<br>**(in Dutch: hoofdonderzoeker/ uitvoerder)**	*Dr. P.M. Snijder, Erasmus MC Department of Neonatal Intensive Care, room Sp4250,*<br>*Dr. Molewaterplein 40, 3015 GD Rotterdam,*<br>*tel. +31650172714, email p.snijder@erasmusmc.nl*

Table of Contents

List of abbreviations and relevant definitions 4
Summary 5
1. Introduction and rationale 6
2. Objective(s) 6
3. Study type 6
4. Study population 8
5. Methods 8
6. Incidental findings 9
7. Statistical analysis 9
8. Ethical considerations 10
9. Handling and storage of data / images / sound recordings / photos / film recordings 10
10. Handling and storage of human material 10
11. Exchange, sharing or transfer of data and/or human material and/or images 10
12. Amendments 11
13. End of study report 11
14. Publication 11
15. References 11
16. Attachments .... **Fout! Bladwijzer niet gedefinieerd.**

List of abbreviations and relevant definitions*

**CTA**	Clinical Trial Agreement
**De novo biobank**	A new data, human material or imaging collection
**DMP**	Data Management Plan
**DPIA**	Data Protection Impact Assessment
**DTA**	Data Transfer Agreement
**Exception consent**	Form Care for data Template, in Dutch: Formulier uitzondering toestemming
**GCP**	Good Clinical Practice
**GDPR**	General Data Protection Regulation in Dutch: Algemene Verordening Gegevensbescherming
**IC**	Informed Consent
**IFU**	Instruction For Use
**MTA**	Material Transfer Agreement
**NWTC**	Non-WMO Review Committee; in Dutch: Niet WMO Toetsingscommissie
**UAVG**	Dutch Act on Implementation of the General Data Protection Regulation; in Dutch: Uitvoeringswet Algemene Verordening Gegevensbescherming
**WMO**	Medical Research Involving Human Subjects Act, in Dutch: Wet Medisch-wetenschappelijk Onderzoek met Mensen

Summary

Rationale
Fetal haemoglobin (HbF) predominates during fetal life and has a higher oxygen affinity than adult haemoglobin (HbA). In preterm neonates, transfusion with adult donor red blood cells may accelerate the postnatal decline in circulating HbF. Lower HbF levels have been associated with prematurity-related diseases, but gestational age-specific HbF values at birth and longitudinal postnatal HbF trajectories remain incompletely characterised. Better characterisation of these patterns is needed to understand postnatal HbF dynamics and their relationship with transfusion exposure and clinical outcomes.

Objective(s)
The primary objectives are to establish gestational age-specific HbF values at birth in neonates born between 24 and 42 weeks of gestation and to characterise longitudinal postnatal HbF trajectories in neonates born before 30 weeks of gestation. Secondary objectives are to investigate the association of red blood cell transfusion exposure and prematurity-related outcomes with longitudinal HbF trajectories.

Study type
Single-centre retrospective observational cohort study using routinely collected clinical and laboratory data.

Study population
Neonates born between 24 and 42 weeks of gestation and admitted to the NICU of Erasmus MC Sophia Children's Hospital between February 2020 and February 2024 with available HbF measurements. Longitudinal analyses will be performed in neonates born before 30 weeks of gestation.

Methods
Routinely collected HbF measurements from blood gas analyses will be combined with clinical data obtained from the neonatal database and electronic patient records. Gestational age-specific HbF values at birth and longitudinal postnatal HbF trajectories will be evaluated. In neonates born before 30 weeks of gestation, RBC transfusion exposure and prematurity-related outcomes will be assessed in relation to longitudinal HbF trajectories using appropriate longitudinal statistical methods.

Burden and risks
There is no additional burden or risk to participants because the study exclusively uses data collected during routine clinical care and requires no additional measurements, interventions, or study visits.

Recruitment and consent
No additional recruitment procedures will be performed. The study uses existing clinical data from neonates whose parents provided consent for the use of their child's data for research.

1. Introduction and rationale

Extremely preterm neonates, born before 28 weeks of gestation, are exposed to the extrauterine environment before their organs have fully matured. Neonatal intensive care is aimed at recreating intrauterine conditions as closely as possible to support organ development. The development of organs depends on a balanced supply of oxygen, as both hypoxia and hyperoxia, may have detrimental effects on neonatal clinical outcomes.[1-5]

Oxygen delivery to tissues is mediated by the oxygen-carrying protein hemoglobin (Hb) in red blood cells (RBCs). During fetal life and the neonatal period, this is predominantly fetal hemoglobin (HbF) consisting of two α- and two γ-chains. This HbF has a high oxygen affinity and a low affinity for 2,3-bisphosphoglyxerate (2,3-BPG), shifting the .[6, 7] the oxygen-hemoglobin dissociation curve to the left. This enables the transfer of oxygen from the maternal to the fetal circulation the low oxygen saturated environment. After term birth, HbF is gradually replaced by adult hemoglobin (HbA), consisting of two α- and two β-chains. HbA binds 2,3-BPG strongly due to a high affinity, thereby facilitating oxygen release to tissues.[6] By 12 months of age, HbA typically accounts for 98% of total Hb in children.[8]

In very premature neonates, recent findings suggest, that this gradual HbF-HbA switch may be accelerated by RBC transfusions. As these are derived from adult donors, they mainly contain HbA. This will lead to a transfusion-induced replacement of HbF by HbA and forces the dissociation curve to shift to the right in a short time-interval, and therefore, may result in an increased oxygen delivery to immature tissues in comparison with HbF.[9] As these tissues are not yet well-equipped to manage excessive free oxygen radicals, increased oxygen release may cause oxidative stress.[10] Both a sharp decline in HbF and RBC transfusions have been associated in a few case-series with the development of the prematurity related diseases (PRDs) retinopathy of prematurity (ROP), bronchopulmonary dysplasia (BPD) and necrotizing enterocolitis (NEC).[2-4, 11] In a series of longitudinal studies in a Swedish cohort of >400 neonates born <30 weeks (2009–2015), low HbF fractions during the first postnatal week were associated with higher FiO_2 and alveolar–arterial gradients during admission as well as with increased risk of ROP and BPD at follow-up.[3, 12, 13] These findings suggest that transfusion-induced HbF decline may contribute to oxidative stress and pulmonary or retinal injury.

These results have been the start of a growing interest in HbF-preserving strategies, as prevention of transfusion-induced HbF decline may positively impact the outcome of these already vulnerable premature neonates. One such novel strategy proposed, is the development of cord blood-derived RBCs from healthy term neonates as an alternative RBC-transfusion source. Naturally, these contain high HbF levels. However, , an understanding of normal HbF ranges, as well as postnatal dynamics of HbF in very premature neonates is required.

2. Objective(s)

Primary objectives:

To establish gestational age-specific HbF values at birth in neonates born between 24 and 42 weeks of gestation.
To characterise longitudinal postnatal HbF trajectories in neonates born before 30 weeks of gestation.

Secondary objectives:
To investigate the association of red blood cell transfusion exposure and prematurity-related outcomes with longitudinal HbF trajectories in neonates born before 30 weeks of gestation.

3. Study type

3.1. Study type

☒ Retrospective

☐ Prospective
☐ Combination Retrospective/Prospective

3.2. **Single center / Multicenter**

☒ Single center
☐ Multicenter

3.3 **Check all the applicable boxes**

☒ Medical records (re-use of data from healthcare, including AI)
☐ Case report
☐ Re-use data from research
☐ Evaluations of quality of healthcare (retrospective
☐ Research with additional use of residual material from regular healthcare
☐ Research with re-use of human material from research or existing biobank
☐ De novo biobank
☐ Phase IV research
☐ Healthcare evaluation research (prospective)
☐ Research with medical devices
☐ Research with In Vitro Diagnostic Tests
☐ Other research, describe

4. Study population

4.1. Study population

☐ Adults (16 years and older)

☒ Minors (younger than 16 years)

☐ Incapacitated adults (16 years and older)

☐ Incapacitated minors (younger than 16 years)

4.2. Population (base)

Neonates born between 24 and 42 weeks of gestation who were admitted to the neonatal intensive care unit of Erasmus MC Sophia Children's Hospital, Rotterdam, the Netherlands, between February 2020 and February 2024 and had HbF measurements available as part of routine clinical care.

4.3. Inclusion criteria

For the gestational age-specific HbF analysis, neonates born between 24 and 42 weeks of gestation with an HbF measurement obtained on postnatal day 0 were included. For longitudinal analyses, neonates born before 30 weeks of gestation with at least one available HbF measurement were included.

4.4. Exclusion criteria

All neonates whose parents who did not give consent for the child's data to be used.

4.5. Sample size calculation

All eligible neonates meeting the inclusion and exclusion criteria during the study period will be included. As the study includes the complete eligible cohort rather than a sampled population, no formal a priori sample size calculation was performed.

5. Methods

5.1. Research methods

Routinely collected clinical and laboratory data from eligible neonates admitted to the NICU during the study period will be retrospectively obtained from the available neonatal database and electronic patient records. HbF measurements obtained as part of routine blood gas analyses will be used to determine gestational age-specific HbF values at birth and to characterise the postnatal HbF course.

For neonates born before 30 weeks' gestation, additional clinical data will be collected, including gestational age, birth weight, sex, Apgar score, mode of delivery, length of NICU stay, RBC transfusion exposure, and prematurity-related outcomes. RBC transfusions occurring between NICU admission and the last available HbF measurement will be recorded. Prematurity-related outcomes will include retinopathy of prematurity, bronchopulmonary dysplasia, necrotizing enterocolitis, and death before discharge.

No additional measurements, interventions, or study visits will be performed for the purpose of this study.

5.2. Standard clinical care versus extra for research
Not applicable.

5.3. Burden and risks
Not applicable.

5.4. Medical device(s) / In vitro diagnostic tests
Not applicable.

6. Incidental findings

6.1. Chance of incidental findings
Is there a chance of incidental findings?

☐ Yes

☒ No

6.2. Procedures
Not applicable.

7. Statistical analysis

7.1 Main study parameters/endpoints

To establish gestational age-specific HbF values at birth in neonates born between 24 and 42 weeks of gestation.

To characterise longitudinal postnatal HbF trajectories in neonates born before 30 weeks of gestation.

7.2 Secondary study parameters/endpoints

To investigate the association of red blood cell transfusion exposure and prematurity-related outcomes with longitudinal HbF trajectories in neonates born before 30 weeks of gestation.

7.3 Other study parameters
Not applicable.

7.4 Analysis
Statistical analyses will be performed using R. Descriptive statistics will be used to summarize clinical and laboratory characteristics. Continuous variables will be presented as mean with standard deviation (SD) or median with interquartile range (IQR), depending on their distribution. Categorical variables will be presented as numbers and percentages. A p-value $<0{\cdot}05$ will be considered statistically significant.

Gestational age-specific HbF values at birth will be evaluated using HbF measurements obtained on postnatal day 0. Postnatal HbF trajectories will be described according to postnatal age and gestational age, using appropriate graphical and longitudinal methods.

RBC transfusion exposure will be described according to gestational age, including the timing and number of transfusions. The association between RBC transfusion exposure and longitudinal HbF trajectories will be evaluated using mixed-effects modelling to account for repeated HbF measurements within individual neonates.

Differences in longitudinal HbF trajectories according to prematurity-related disease status will be explored using mixed-effects modelling.

8. Ethical considerations

8.1 Regulation statement

The study will be conducted according to the principles of the Declaration of Helsinki (version, date, see for the most recent version: www.wma.net), Gedragscode Gezondheidsonderzoek 2022 and in accordance with other guidelines, regulations and Acts (if applicable, please specify).

8.2 Informed consent

Will the subjects be asked for informed consent?

☐ Yes *(Upload Participant Information Letter and Informed Consent)*

☒ No, consent already given in previous study *(Upload Participant Information Letter and Informed Consent previous study)*

☐ No, this research will be performed under the exception consent *(Upload form Care for data Template, in Dutch: Formulier uitzondering toestemming)*

☐ Other (e.g. partly, indirectly) *Please describe the situation.*

8.3 Recruitment and informed consent procedures

Not applicable.

9. Handling and storage of data / images / sound recordings / photos / film recordings

The study will be conducted in accordance to the principles of the Declaration of Helsinki version 2013 (www.wma.net), and the investigators comply with the principles enunciated in this declaration. The data is coded (patients will be numbered) and only the investigators are able to access the data. Only the primary investigator will have access to the study key.

10. Handling and storage of human material

Not applicable.

11. Exchange, sharing or transfer of data and/or human material and/or images

Not applicable.

12. Amendments

Amendments are changes made to the research after a favorable opinion by the NWTC has been given.

All amendments must be submitted to the NWTC that gave the favorable opinion.

Substantial amendments must be approved by the Niet WMO Toetsingscommissie before they can be implemented.

13. End of study report

Within one year after the end of the study a final study report will be submitted with the results of the study, including any publications/abstracts of the study.

14. Publication

Do you have the intention to submit the study results in a manuscript for publication in a journal:

☒ Yes

☐ No, *please motivate*

15. References

1 Bednarczuk N, Williams EE, Kaltsogianni O, Greenough A, Dassios T. Postnatal temporal changes of foetal haemoglobin in prematurely born infants. Acta Paediatrica. 2022; 111: 1338-40.

2 Stutchfield CJ, Jain A, Odd D, Williams C, Markham R. Foetal haemoglobin, blood transfusion, and retinopathy of prematurity in very preterm infants: a pilot prospective cohort study. Eye. 2017; 31: 1451-5.

3 Hellström W, Martinsson T, Hellstrom A, Morsing E, Ley D. Fetal haemoglobin and bronchopulmonary dysplasia in neonates: an observational study. Archives of Disease in Childhood - Fetal and Neonatal Edition. 2021; 106: 88-92.

4 Hellström W, Martinsson T, Morsing E, Gränse L, Ley D, Hellström A. Low fraction of fetal haemoglobin is associated with retinopathy of prematurity in the very preterm infant. British Journal of Ophthalmology. 2022; 106: 970-4.

5 Falsaperla R, Lombardo F, Filosco F, Romano C, Saporito MAN, Puglisi F, et al. Oxidative Stress in Preterm Infants: Overview of Current Evidence and Future Prospects. Pharmaceuticals. 2020; 13: 145.

6 Pellegrino C, Stone EF, Valentini CG, Teofili L. Fetal Red Blood Cells: A Comprehensive Review of Biological Properties and Implications for Neonatal Transfusion. Cells. 2024; 13: 1843.

7 Pritišanac E, Urlesberger B, Schwaberger B, Pichler G. Fetal Hemoglobin and Tissue Oxygenation Measured With Near-Infrared Spectroscopy—A Systematic Qualitative Review. Frontiers in Pediatrics. 2021; 9.

8 Wilber A, Nienhuis AW, Persons DA. Transcriptional regulation of fetal to adult hemoglobin switching: new therapeutic opportunities. Blood. 2011; 117: 3945-53.

9 Halleux VD, Truttmann A, Gagnon C, Bard H. The effect of blood transfusion on the hemoglobin oxygen dissociation curve of very early preterm infants during the first week of life. Seminars in Perinatology. 2002; 26: 411-5.
10 Lembo C, Buonocore G, Perrone S. Oxidative Stress in Preterm Newborns. Antioxidants. 2021; 10: 1672.
11 Patel RM, Knezevic A, Shenvi N, Hinkes M, Keene S, Roback JD, et al. Association of Red Blood Cell Transfusion, Anemia, and Necrotizing Enterocolitis in Very Low-Birth-Weight Infants. JAMA. 2016; 315: 889.
12 Hellström W, Martinsson T, Morsing E, Gränse L, Ley D, Hellström A. Low fraction of fetal haemoglobin is associated with retinopathy of prematurity in the very preterm infant. Br J Ophthalmol. 2022; 106: 970-4.
13 Ulinder T, Hellström W, Gadsbøll C, Nilsson L, Gebka M, Robertz G, et al. Fetal haemoglobin and oxygen requirement in preterm infants: an observational study. Archives of Disease in Childhood - Fetal and Neonatal Edition. 2025; 110: 285-90.
